# Editorial: Digital technology in the diagnosis and treatment of osteoporotic fractures in the elderly

**DOI:** 10.3389/fmed.2025.1638663

**Published:** 2025-06-20

**Authors:** Jun Hu

**Affiliations:** The First Affiliated Hospital With Nanjing Medical University, Nanjing, China

**Keywords:** digital technology, osteoporotic, fracture, elderly, diagnosis, treatment, aging

Osteoporotic fractures, especially hip fractures, represent a significant public health issue affecting the aging global population (1, 2). Despite considerable progress in surgical techniques and perioperative care, high morbidity and mortality persist, underscoring the need for innovative, data-driven strategies. The integration of digital technologies into clinical practice offers promising solutions for improved early detection, risk stratification, and tailored treatment approaches (3).

**Figure 1:**
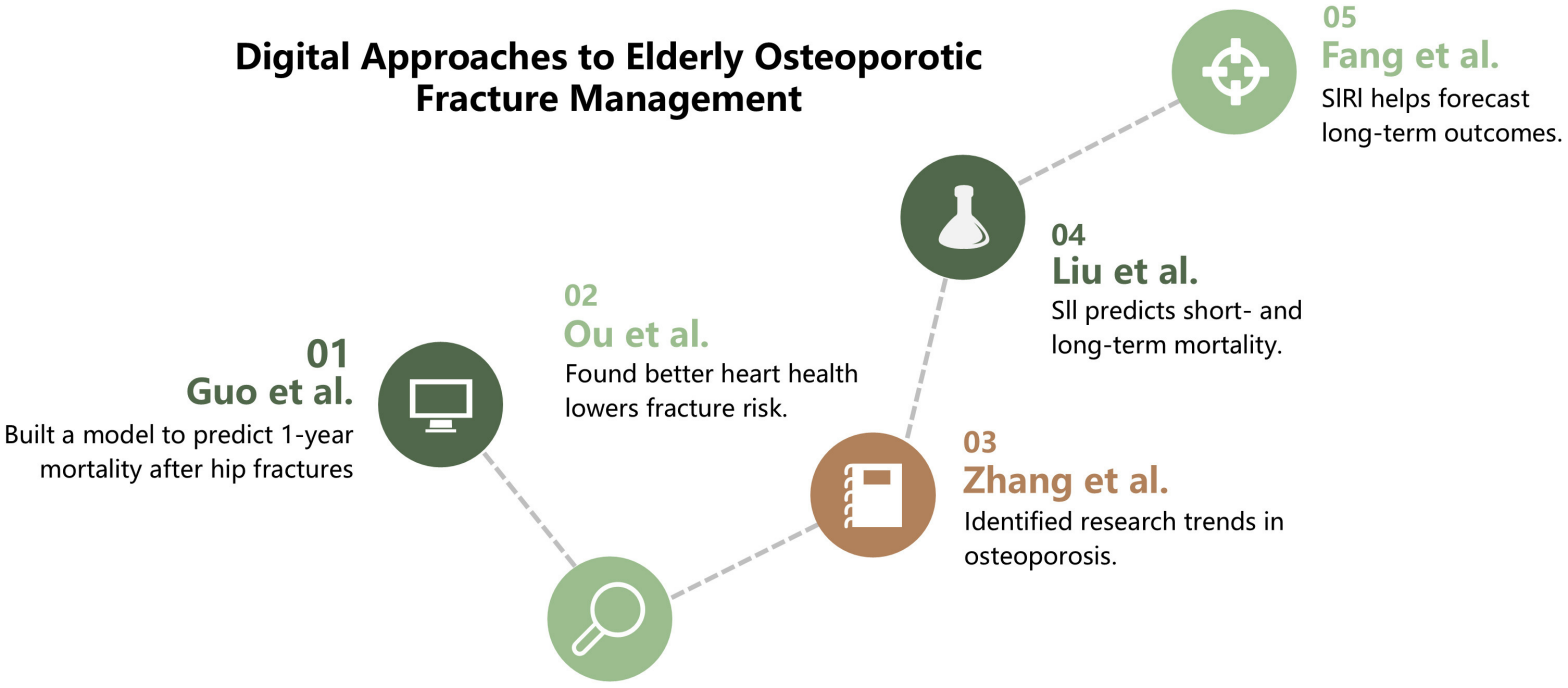
Overview of thematic contributions within the Research Topic. This figure summarizes the main focus of each included study, highlighting how diverse digital approaches—ranging from predictive modeling to epidemiological and bibliometric analyses—contribute to the overall advancement of osteoporotic fracture management in the elderly.

This Research Topic, titled “*Digital technology in the diagnosis and treatment of osteoporotic fractures in the elderly*,” aims to showcase how cutting-edge digital technologies can significantly enhance the management of osteoporotic fractures. The five featured studies explore various critical dimensions, including predictive modeling, epidemiology, biomarker research, and bibliometric analytics. Figure 1 summarizes the thematic integration, illustrating how digital innovations collectively enable comprehensive fracture management in geriatric care.

Guo et al. present a robust predictive nomogram designed to evaluate one-year mortality risk following hip fractures among older adults. Utilizing advanced statistical modeling (LASSO regression), the authors identified essential predictive factors such as patient age, comorbidities, surgical intervention status, and biochemical indicators like hemoglobin and renal function. This clinically relevant digital tool provides clinicians with the means to promptly identify high-risk individuals, thereby enabling targeted interventions and enhanced patient outcomes.

Ou et al. conducted an extensive epidemiological investigation using data from the U.S. NHANES database, examining the link between cardiovascular health, quantified by the Life's Essential 8 (LE8) metrics, and osteoporotic fracture incidence. Their findings suggest a substantial inverse association, highlighting that superior cardiovascular health markedly reduces fracture risk. These insights advocate for comprehensive health strategies, suggesting integrated preventive programs addressing cardiovascular and bone health simultaneously could markedly mitigate fracture incidence.

Zhang et al. deliver a meticulous bibliometric analysis covering a decade of osteoporosis research. By applying sophisticated informatic tools such as CiteSpace and VOSviewer, the authors identified key global research trends and emerging focal areas, including osteoblast biology, sarcopenia, gut microbiome, and therapeutic advancements such as denosumab. This analytical approach effectively delineates existing knowledge gaps and directs future research agendas, emphasizing interdisciplinary collaboration and strategic scientific focus.

Two additional studies emphasize inflammation-based biomarkers as prognostic indicators derived from routine laboratory data. Liu et al. investigated the Systemic Immune-Inflammation Index (SII) in critically ill elderly hip fracture patients utilizing data from the MIMIC-IV database, demonstrating its effectiveness in predicting both short-term (30-day) and long-term (one-year) mortality. Similarly, Fang et al. conducted a retrospective study on the Systemic Inflammation Response Index (SIRI) across a 10-year cohort, affirming its predictive value for long-term mortality outcomes. Both studies underscore the clinical relevance of inflammation indices as accessible digital biomarkers for early identification of high-risk patients and tailored clinical interventions.

Collectively, these studies underscore the transformative potential of digital technology in osteoporotic fracture management. Through advanced analytics, comprehensive data analysis, and interdisciplinary cooperation, digital solutions enable precision medicine approaches, significantly enhancing the quality and outcomes of patient care. Moving forward, critical future directions include the validation of these predictive models across diverse populations and clinical settings, alongside their integration into real-world clinical workflows. Prospective clinical trials are necessary to rigorously assess the effectiveness of these digital tools. Additionally, advancing AI-driven diagnostic technologies and digital platforms for patient monitoring and rehabilitation represents promising avenues for research and clinical application (4). International collaboration will be essential in promoting data-sharing initiatives and multicenter studies, ensuring the broad applicability and global accessibility of digital innovations (5). Ultimately, sustained collaborative efforts will elevate standards of care, delivering significant improvements in health outcomes for elderly patients experiencing osteoporotic fractures worldwide.
